# Harlequin Ichthyosis: A Fatal Case Report in Al-Medina, Saudi Arabia

**DOI:** 10.7759/cureus.23533

**Published:** 2022-03-27

**Authors:** Omar Shahada, Ahmed Kurdi, Duaa Al ahmadi

**Affiliations:** 1 Dermatology, King Salman bin Abdulaziz Medical City, Medina, SAU; 2 Dermatology, Alrayan Medical College, Medina, SAU

**Keywords:** saudi arabia, medina, abca12 gene mutation, autosomal recessive, consanguinity, genetic, collodion, lamellar, ichthyosis, harlequin

## Abstract

Harlequin ichthyosis (HI) is an autosomal recessive disorder. It is a fatal disease and many infants born with HI die shortly after birth. The incidence is extremely rare and is reported to be about 1 in 300,000 births. The hallmark of the disease is alligator-like horned skin that is severely keratinized. Several cases of fetal HI have been reported, but to contribute to the collective knowledge of this rare severe skin disorder, we report the first case, from Medina, Saudi Arabia, of a 45-year-old woman who delivered a newborn infant with HI and has a previous history of six infants who died from a similar condition. Obtaining a prenatal diagnosis, in this case, is critical to alleviate the physical and mental suffering experienced by parents and relatives. Management is mainly supportive until now, as no curable therapy has been proven. Genetic counseling of the ABCA12 gene is advised in consanguinity marriage with positive family history.

## Introduction

Harlequin ichthyosis (HI) is an autosomal recessively inherited disorder; it is the most severe form of congeni1tal ichthyosis. Newborn with this condition present with severe thickening of keratin layer, and shiny-plate-like scaling of the skin separated by erythematous fissures [1]. In addition, the newborn suffers from severe eclabium and ectropion with hypoplastic digits and incomplete development of eyes, nose, and ears. There is also irregular control of body temperature and limited movement of the extremities [2].

HI has a high mortality rate due to disease complications mainly secondary to infection from fissuring of the hyperkeratotic plates, and respiratory failure as a result of restricted chest wall movement by the effect of rigid skin [3].

Consanguinity is thought to be an important risk factor for HI [4], as it has been linked to an autosomal recessive inheritance of a mutation in ABCA12 gene, which is generally responsible for lipid transport in keratinocytes and is necessary for lipid transport into lamellar granules during the formation of the lipid barrier [5].

## Case presentation

**Figure 1:**
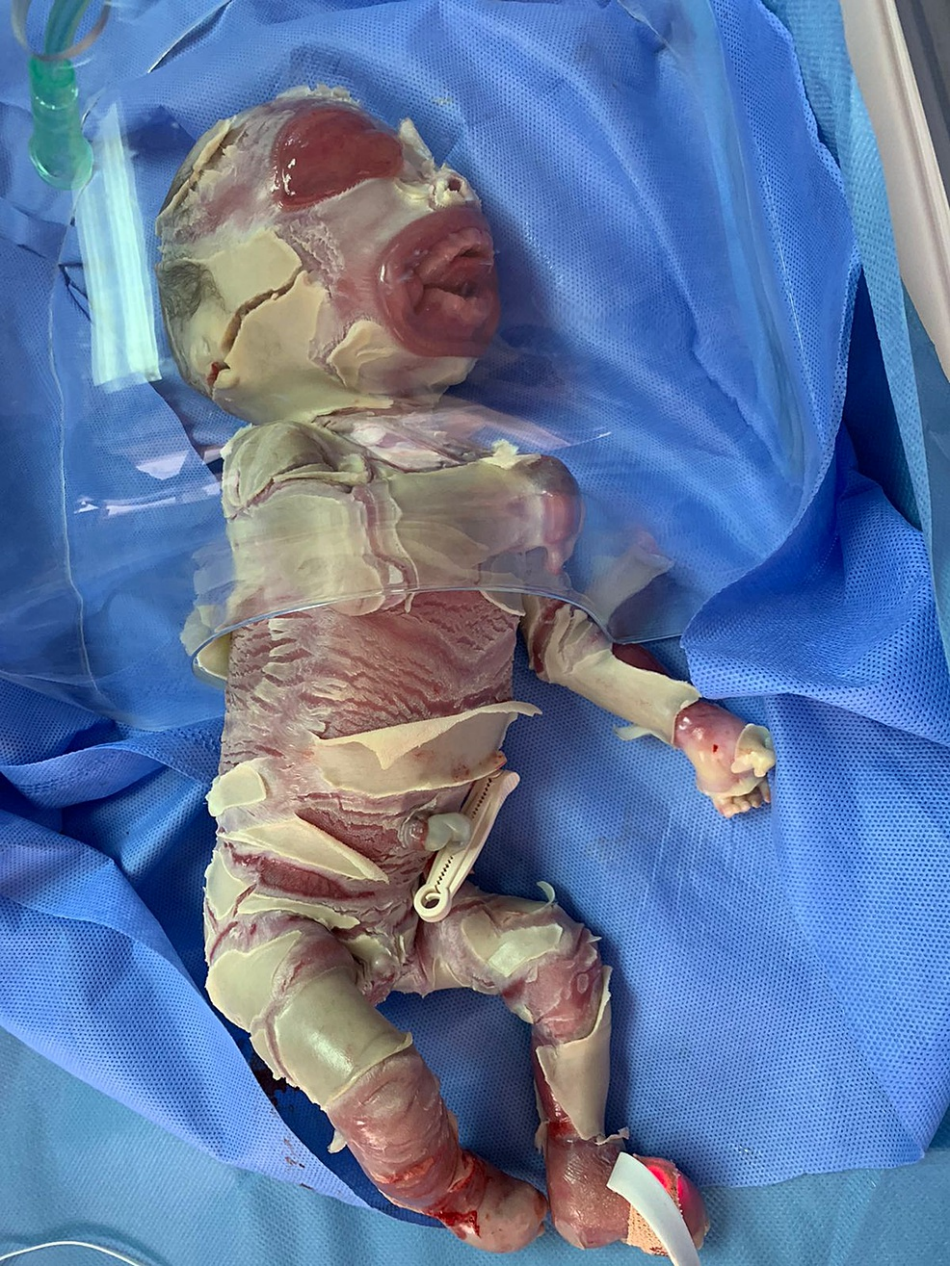
Baby with severe harlequin ichthyosis.

**Figure 2:**
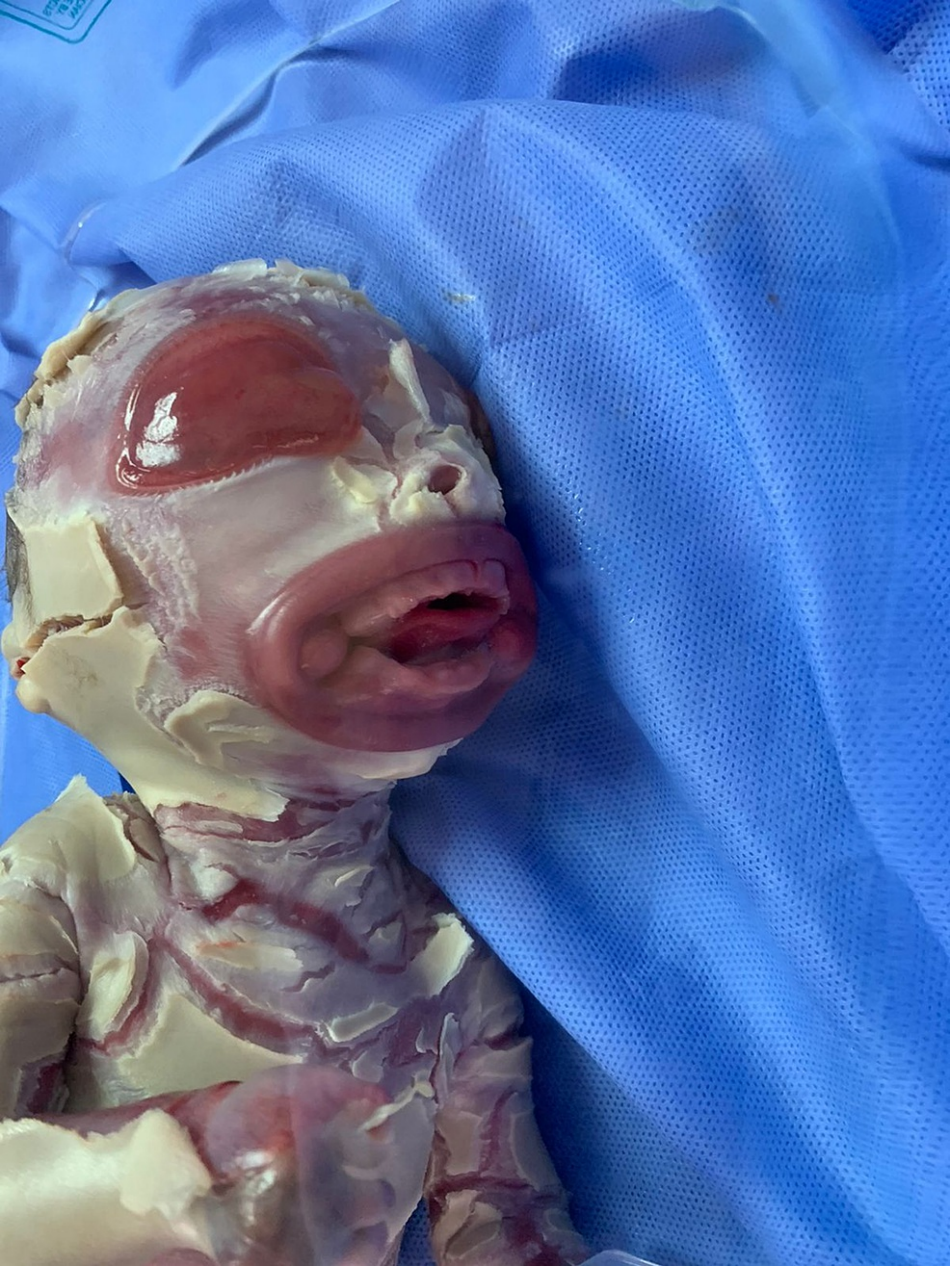
Severe cracked and deep fissure, thick skin with hyperkeratinization, ectropion, eclabium, flatted nose, wide mouth, deformed eyes and ears.

**Figure 3:**
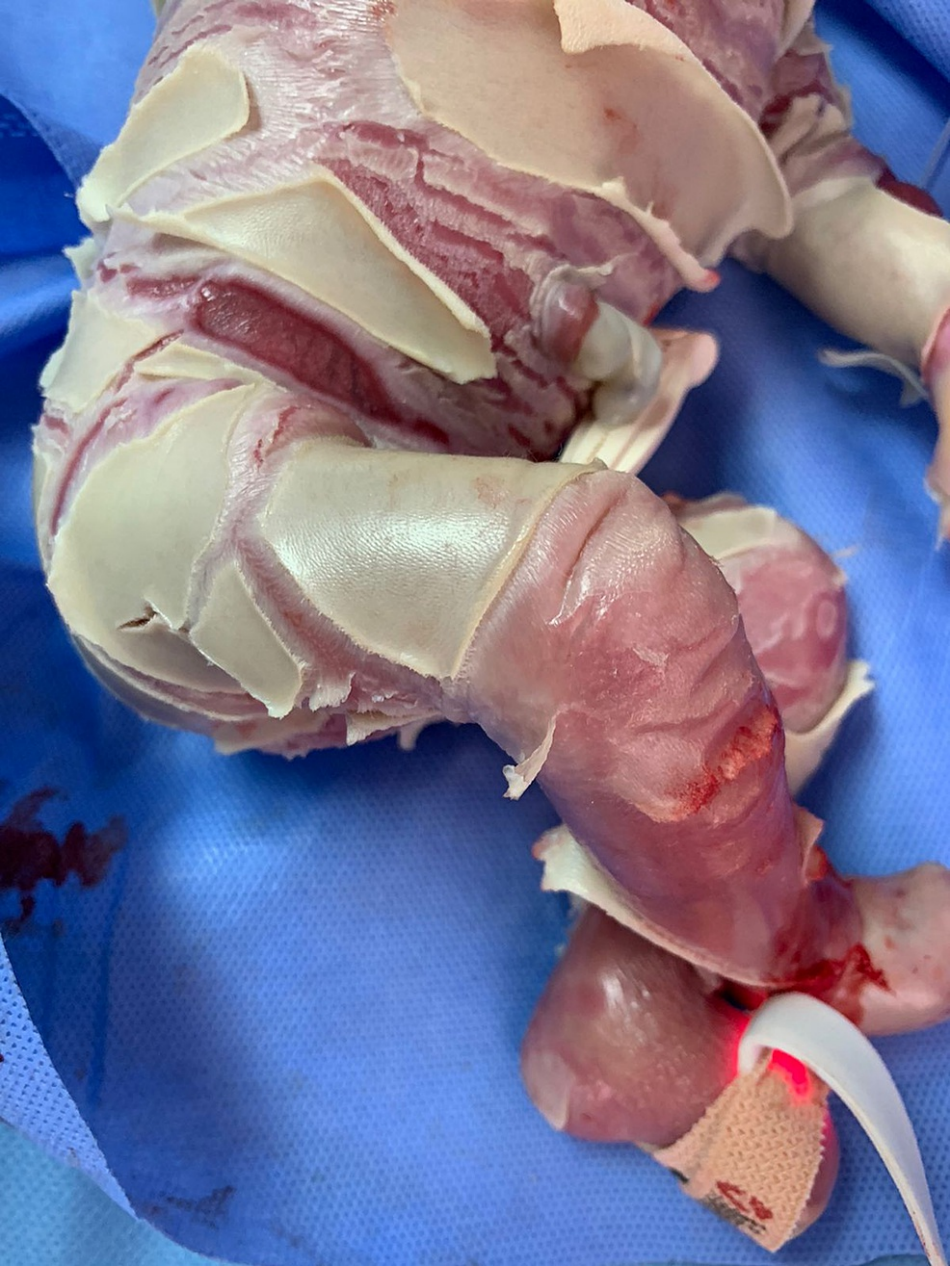
Abdomen and lower limbs of the patient with harlequin ichthyosis.

A 39-week unbooked full-term Afghani male was born with a collodion membrane. The birth was uneventful with spontaneous vaginal delivery and weighed 1.6 kg. The mother was 45 years old, Gravida 11 Parity 7 +3 L4. Since she does not have health insurance, she did not attend any screening clinics. The parents had a close relation with six infants who died within one to two days of their birth because of a similar condition. Four other children lived normally. On examination, the baby looked unwell, cyanosed, and vitally unstable with bradycardia (66 beats per minute), bradypnea (18 breaths per minute). Severe cracked and deep fissure, thick skin with hyperkeratinization, ectropion, eclabium, flatted nose, wide mouth, deformed extremities, eyes and ears were noticed (Figures 1-3).

**Figure 4:**
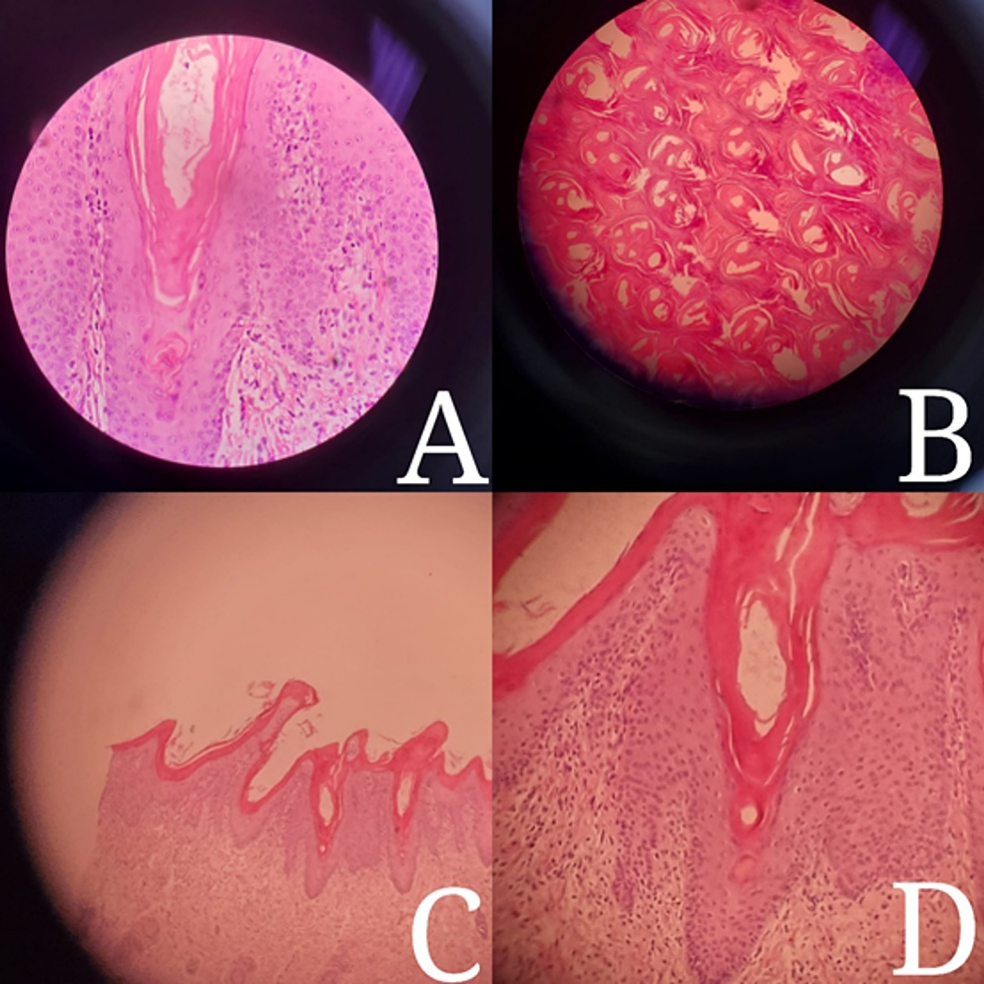
(A) Epidermal hyperplasia, (B) severe hyperkeratosis, (C) thick stratum corneum, loss of basket weave pattern, (D) inflammatory cells infiltration in dermo-epidermal junction. Hematoxylin and eosin stain was used. Magnification power in A and D: 20X, C: 10X, B: 40X

The neonatologist diagnosed the baby with multiple congenital anomalies including cardiac, lung and renal anomalies. The management started in the neonatal intensive care unit (NICU) with excessive emollients and broad-spectrum antibiotics. After being born, the baby lived for 35 hours before dying of respiratory distress. Education and genetic counseling were given to the parents to prepare them for future pregnancies. A punch biopsy of 4 mm was taken deeply from his right thigh a day before he passed away with written consent from the parents. The examination of the skin biopsy specimen showed a section of the epidermis and dermis. The epidermis showed increased stratum corneum with parakeratosis, focal hypergranulosis, loss of normal basket weave pattern, few inflammatory cells infiltration consisting of lymphocytes in the dermo-epidermal junction (Figure 4A-4D).

## Discussion

The most severe form of autosomal recessive congenital ichthyosis is harlequin ichthyosis, which is marked by a significant thickening of keratin in the fetal skin. At birth, the affected fetus is born with a large, horny shell of dense, plate-like scales separated by erythematous fissures and other abnormalities of the face including:

1) Presence of severe ectropion in the eyes.

2) Flattening of ears with the absence of retroauricular folds.

3) Eclabium which is caused by severe traction on the lips and by a fixed open mouth, which may compromise the ability to feed.

4) The patient manifests nasal hypoplasia with eroded nasal alae, and obstructed nares.

The limbs are covered with thick hyperkeratotic skin, resulting in a poor range of mobility [5]. In addition, due to limited chest expansion and physical abnormalities, patients with HI may experience respiratory failure. Furthermore, feeding problems can cause complications such as low blood sugar, dehydration, and kidney failure. Moreover, temperature dysregulation and secondary infection would occur [6].

A mutation in the lipid-transporter gene ABCA12 on chromosome 2 is the underlying genetic anomaly in harlequin ichthyosis. This gene is important for the transfer of lipids to epidermal cells and the appropriate development of the skin [7].

It is crucial to have a perinatal diagnosis. An ultrasound of the baby's mouth, primarily at the 17th week of pregnancy, and an examination of amniotic fluid cells have been both demonstrated to produce definitive results. A skin biopsy would likely reveal structural anomalies of lamellar granules and epidermal keratin expression in a postnatal diagnosis. However, the overall clinical appearance of the infant is usually enough to make a diagnosis. Moreover, a full explanation of family history, consanguinity between parents, information regarding past pregnancies, and whether or not other children had any underlying dermatological problem are all important considerations [8]. In this case, there was a previous family history of six similar newborns who suffered and died within a day or two of their birth. Therefore, a genetic complete sequence analysis for mutations in the ABCA12 gene must be performed.

The mortality rate of HI is high. According to previous reports, the newborn mortality rate is around 50%, and many infants with HI die soon after birth. However, improved neonatal intensive care and early therapy with systemic retinoids may increase survival. Although, the kind of mutation affects survival; patients with the compound heterozygote mutation have a higher chance of surviving than those with the homozygote mutation [9].

Initial management includes securing the patient's airway, breathing, and circulation stability. Broad-spectrum antibiotics are given to prevent secondary bacterial infection, as well as emollients that soften the skin, particularly those containing urea, salicylic acid, or alpha hydroxy acids, are highly effective [8]. Early intubation may be necessary [9]. Nutritional support with tube feeds is required until eclabium resolves and infants can start nursing. Ophthalmology consultation can assist with the early treatment of ectropion, which is noticeable at first but diminishes as the scale is shed. Liberal application of petrolatum is needed several times per day. Furthermore, debridement of hyperkeratosis constrictive bands should be done carefully to avoid digital ischemia [3].

## Conclusions

We conclude that this patient's clinical and pathological abnormalities are aligned with harlequin ichthyosis, which is a life-threatening rare genetic disorder that presents with several complications that lead to poor prognosis and high mortality rate, as this patient lived for 35 hours then passed away. We recommend in cases of consanguinity marriage with a positive family history of HI to conduct genetic counseling for the ABCA12 gene. Since there is no cure, management is primarily supportive.

Several cases of children with HI have been reported, but according to our knowledge, the current study is the first one in Al-Medina, Saudi Arabia.
